# *Bifidobacterium* in anticancer immunochemotherapy: friend or foe?

**DOI:** 10.20517/mrr.2023.23

**Published:** 2023-07-10

**Authors:** Giorgia Procaccianti, Sara Roggiani, Gabriele Conti, Patrizia Brigidi, Silvia Turroni, Federica D’Amico

**Affiliations:** ^1^Unit of Microbiome Science and Biotechnology, Department of Pharmacy and Biotechnology, University of Bologna, Bologna 40126, Italy.; ^2^Microbiomics Unit, Department of Medical and Surgical Sciences, University of Bologna, Bologna 40138, Italy.

**Keywords:** Bifidobacterium, anti-inflammatory, pro-inflammatory, lactate, cancer, immunochemotherapy, response to therapy

## Abstract

The gut microbiome has received a crescendo of attention in recent years due to myriad influences on human pathophysiology, including cancer. Anticancer therapy research is constantly looking for new hints to improve response to therapy while reducing the risk of relapse. In this scenario, Bifidobacterium, which inhabits the gut microbial ecosystem (especially that of children) and is considered a health-associated microbe, has emerged as a key target to assist anticancer treatments for a better prognosis. However, some researchers have recently hypothesized an unfavorable role of Bifidobacterium spp. in anticancer immunochemotherapy, leading to some confusion in the field. This narrative review summarizes the current knowledge on the role of Bifidobacterium spp. in relation to anticancer treatments, discussing the pros and cons of its presence in the gut microbiome of cancer patients. The current intervention strategies based on the administration of probiotic strains of Bifidobacterium are then discussed. Finally, the need to conduct further studies, especially functional ones, is underlined to provide robust experimental evidence, especially on the underlying molecular mechanisms, and thus resolve the controversies on this microbe for the long-term success of immunochemotherapy.

## INTRODUCTION

*Bifidobacterium* are non-spore-forming Gram-positive bacteria with a bifurcated (“bifid”) shape, belonging to the Actinomycetota phylum. Since 1900, when they were first isolated from the feces of breastfed infants^[1]^, about 118 species have been identified and included in the Bifidobacterium genus^[2]^ (lpsn.dsmz.de). Over the years, species belonging to this genus have become of increasing relevance given their beneficial effects on human health, so much so as to obtain the GRAS (Generally Recognized As Safe) status by the Food and Drug Administration (FDA) and inclusion in the QPS (Qualified Presumption of Safety) list of the European Food Safety Authority (EFSA), and be widely used in the food and pharmaceutical industry as probiotics.

*Bifidobacterium* can be found in several ecological niches, including the gastrointestinal tract of humans and other mammals, insects, and birds^[3,4]^. Bifidobacterium spp. are the first colonizers of the gut microbiota (GM) of infants, in particular of those breastfed, where they abound due to their ability to metabolize human milk oligosaccharides (HMOs)^[5]^. Later, with the cessation of breastfeeding and the introduction of solid foods, the near monodominance of Bifidobacterium spp. comes to an end and the GM begins to be colonized by other (more strictly anerobic) microorganisms, assuming a more adult-like composition^[6]^. In most adult-type GMs, Bifidobacterium still constitutes a relevant component, albeit in much lower proportions (mean relative abundance, 2-14%)^[7]^. Further decline of Bifidobacterium spp. can be observed with aging, along with a decrease in species diversity, which has been correlated with immunosenescence^[8-10]^. In contrast, a higher occurrence or prevalence of *Bifidobacterium* was found in centenarians (and beyond) worldwide, suggesting an association with healthy aging and longevity^[10-12]^.

The ecological fitness of Bifidobacterium can be explained by its ability to metabolize dietary and host-derived carbohydrates, leading to the production of short-chain fatty acids (SCFAs) with a key and multifactorial role in human physiology^[2,13,14]^. The glycobiome of *Bifidobacterium* is indeed one of the largest among other gut commensals, comprising several glycosyl hydrolases, glycosyltransferases, and carbohydrate esterases^[15]^. Additionally, certain Bifidobacterium spp. can synthesize folates and tryptophan-derived indoles^[16]^. The former are involved in cell metabolism, immune development, and epigenetic modifications^[17-19]^, while the latter improve the integrity of the intestinal epithelium and regulate intestinal mucosa immune responses as aryl hydrocarbon receptor ligands^[20-22]^. *Bifidobacterium* can also limit the growth of pathogens by modulating the immune system and producing antibacterial peptides (i.e., bacteriocins) and organic acids (i.e*.*, lactic and acetic acids)^[23]^. In particular, the accumulation of organic acids leads to acidification of the surrounding environment, thus inhibiting the growth of low pH-sensitive bacteria^[24,25]^.

All these activities underlie the beneficial effects of *Bifidobacterium* spp. and their use as probiotics in different healthcare settings^[26,27]^. Unsurprisingly, GM alterations (i.e., dysbiosis) often include, regardless of age, an underrepresentation of *Bifidobacterium*^[28-31]^. Furthermore, various studies have reported that the administration of *Bifidobacterium* spp., alone or in combination with other lactic acid bacteria (particularly *Lactobacillus*), helps to relieve the symptoms of several intestinal and extraintestinal disorders, e.g., inflammatory bowel disease^[32]^, irritable bowel syndrome^[33]^, antibiotic-associated diarrhea^[34]^ and *Clostridioides difficile*-associated diarrhea^[35]^, necrotizing enterocolitis^[36]^, allergy^[37]^, systemic lupus erythematosus^[38]^, and atopic dermatitis^[39]^.

**Figure 1:**
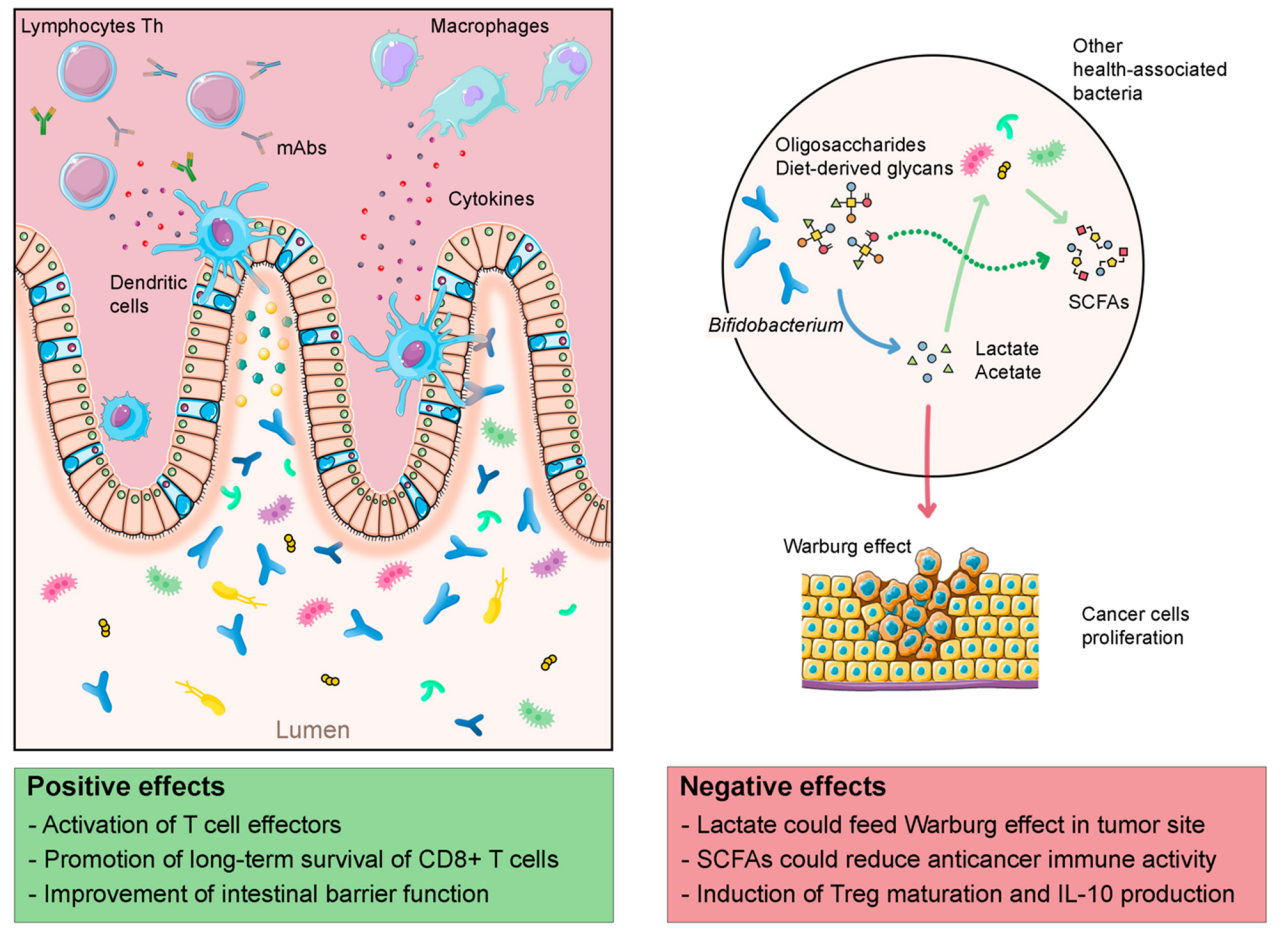
*Bifidobacterium* and its controversial role in the response to anticancer immunochemotherapy. *Bifidobacterium* spp. have been associated with response to immunochemotherapy through stimulation and activation of effector T lymphocytes and dendritic cells. On the other hand, *Bifidobacterium* spp. have been associated with induction of Treg cells, stimulation of anti-inflammatory cytokines, and general immunosuppressive effects (also mediated by short-chain fatty acids – SCFAs). Furthermore, the ability of *Bifidobacterium* to produce lactate suggests a hypothetical link with the “Warburg effect”, known to promote tumor growth, thus potentially liming the effect of immunochemotherapy. The figure was partly generated using Servier Medical Art, provided by Servier, licensed under a Creative Commons Attribution 3.0 unported license and images from Flaticon resources.

While in the aforementioned pathological contexts, the benefits of *Bifidobacterium* spp. are well established, their role in the cancer setting is still controversial, with some evidence suggesting they may improve immune function and reduce postoperative complications, and more recent evidence suggesting an association with immunochemoresistance and treatment failure^[40]^ [Figure 1].

In this review, after summarizing the relationship between GM and immunochemotherapy, we discuss the latest research evaluating the impact of the presence of *Bifidobacterium* in the GM of cancer patients, as well as its administration in probiotic formulations on therapeutic response and key clinical outcomes. Finally, we stress the need to conduct further studies, especially of a functional nature, in order to move from purely associative observations to mechanistic glimpses, which provide robust experimental evidence on the role of *Bifidobacterium* in the anticancer immunochemotherapy landscape. The definition of this role will make it possible to improve current intervention strategies for truly successful precision medicine approaches.

## THE RELATIONSHIP OF THE GUT MICROBIOME WITH RESPONSE TO IMMUNOCHEMOTHERAPY

In recent years, several studies have highlighted a bidirectional relationship between GM and immunochemotherapy in different types of cancer. Indeed, therapeutic efficacy was significantly reduced in the absence of GM, thus suggesting that commensal microbes may modulate anticancer immune responses through several mechanisms^[40,41]^. The first example of this intricate interconnection between gut microbes and anticancer treatment involves cyclophosphamide, an approved chemotherapeutic drug. It has been shown that cyclophosphamide can alter GM composition in mice and promote the translocation of specific Gram-positive bacteria into secondary lymphoid organs, stimulating the production of Th17 cells^[42,43]^. Indeed, germ-free mice or mice treated with broad-spectrum antibiotics showed resistance to cyclophosphamide-based therapies^[43]^. In particular, the antitumor response was associated with increased levels of *Lactobacillus johnsonii*, *Enterococcus hirae* and *Barnesiella intestinihominis*^[43]^. A few years later, elegant work by Daillere *et al.* revealed the mechanisms by which *E. hirae* and *B. intestinihominis* were able to stimulate the immune response against tumor cells^[44]^. The former translocated from the small intestine to secondary lymphoid organs and increased the intratumor CD8/Treg ratio, while the latter was overabundant in the colon where it promoted the infiltration of interferon-gamma (IFN-γ)-producing T cells into cancer lesions. Further confirming these findings, the antitumor activity of cyclophosphamide was restored in murine models receiving an oral gavage of *E. hirae* after antibiotic treatment.

GM can also modulate the efficacy of immunotherapy^[45-47]^. Since their development, immune checkpoint inhibitors have revolutionized the anticancer therapeutic landscape, positively changing the clinical outcomes of several cancers such as melanoma^[48]^ and renal cell carcinoma^[49]^, as well as malignancies considered non-immunogenic such as non-small-cell lung cancer (NSCLC)^[50,51]^ or mismatch-repair-deficient colorectal cancer (CRC)^[52]^. Immune checkpoint therapy targets regulatory pathways in T cells by removing their inhibitory signals, thereby enabling tumor-reactive T cells to unleash an effective antitumor response^[53]^. An early study in antibiotic-treated mice showed altered GM that impaired both CpG-oligonucleotide immunotherapy and platinum-based chemotherapy. On the other hand, the reduction of tumor growth through the production of tumor necrosis factor alpha (TNFα) by myeloid cells and T cells was shown in mice not receiving antibiotics. Indeed, antibiotic treatment impaired the production of TNFα and other cytokines by immune cells including monocytes, macrophages, and dendritic cells, and reduced tumor regression^[42]^. Furthermore, the antitumor effect of anti-cytotoxic T-lymphocyte-associated protein 4 (anti-CTLA-4) antibodies was associated with the presence of distinct *Bacteroides* species that were able to stimulate the T cell response against melanoma. Indeed, germ-free and antibiotic-treated mice did not respond to anti-CTLA-4, while when they were gavaged with *Bacteroides fragilis*, a restoration of the efficacy of the anticancer therapy was observed. The same anticancer outcomes were obtained only by immunizing the same murine models with *B. fragilis* polysaccharides, or by adoptive transfer of *B. fragilis*-specific T cells. In particular, *B. fragilis* was associated with Th1 immune responses in lymph nodes and dendritic cell maturation in the tumor environment, thereby reverting CTLA-4 blockade. Similar results were obtained in melanoma patients in whom anti-CTLA-4 efficacy was associated with T cell responses mediated by an overabundance of *B. fragilis* or *Bacteroides thetaiotaomicron*^[48]^. Fecal microbial transplantation from humans to murine models further confirmed that anti-CTLA-4 treatment favored the outgrowth of *B. fragilis* with all the anticancer properties discussed above. More recently, GM analysis of metastatic melanoma patients receiving ipilimumab, a CTLA-4-targeted immune checkpoint inhibitor, revealed that elevated levels of *Faecalibacterium* and other members of the phylum Firmicutes were associated with not only longer survival but also reduced occurrence of ipilimumab-induced colitis^[54]^. Similarly, programmed cell death protein 1 (PD-1) is one of the inhibitory receptors that downregulate effector functions and suppress the immune response leading to non-activation of the immune cascade against tumors. Immunotherapy against PD-1 allowed to achieve tumor regression in a subset of cancers^[55-59]^. As for GM, studies in renal and lung cancers showed that non-responders were characterized by reduced levels of *Akkermansia muciniphila*, which promoted the recruitment of activated T lymphocytes into the tumor microenvironment via the IL-12 pathway^[47]^. A recent study confirmed the relevance of *Akkermansia* as a prognostic factor for NSCLC patients treated with immune checkpoint inhibitors^[60]^. Specifically, the authors found that baseline relative abundance of *Akkermansia* was linked to therapeutic advantages, namely increased objective response rates and overall survival. Furthermore, the intestinal residence of *Akkermansia* was found to be a proxy for the richness of the gut ecosystem, which is generally related to more favorable outcomes. Finally, two studies in melanoma patients have identified other commensal microbes potentiating the antitumor effects of PD-1 blockade in responders^[45,46]^. Notably, responders exhibited greater interindividual diversity (which suggests retention of one’s GM fingerprint or uniqueness) and higher levels of *Faecalibacterium* and other health-associated *Ruminococcaceae* members, which were involved in enhanced antigen presentation and T cell function in the tumor microenvironment^[45]^. For a list of bacteria other than *Bifidobacterium* that have been suggested to date to play a role in the success (or failure) of immunochemotherapy, please consult the relevant literature (e.g., Table 1 from Gupta *et al.* 2021^[61]^, including pathogenic and non-pathogenic bacteria mediating cancer immunotherapy, and Tang *et al.* 2022^[62]^, who also discussed engineered bacteria with enhanced tumor tropism, significant immunomodulation and improved safety profile, for enhanced therapeutic outcomes in different cancer models).

**Table 1:** Clinical trials registered in the last two years on ClinicalTrials.gov (as accessed in March 2023) involving the use of *Bifidobacterium* spp. as adjuvant therapy in cancer patients. Search terms included “cancer” or “tumor” in combination with “*Bifidobacterium*”

**Title**	**Status**	**Results**	**Condition**	**Intervention**	**Location**	**URL**
Safety and Efficacy of Bifidobacterium Therapy in Patients With Advanced Liver Cancer Receiving Immunotherapy	Recruiting	Not available	Advanced Hepatocellular Carcinoma	*Bifidobacterium bifidum* oral product	China	https://clinicaltrials.gov/ct2/show/NCT05620004
Clinical Study on BIFICO Accelerating Postoperative Liver Function Recovery in Patients With Hepatocellular Carcinoma	Completed	Not available	Hepatocellular Carcinoma	BIFICO (*Bifidobacterium*-based product)	China	https://clinicaltrials.gov/ct2/show/NCT05178524
Lactobacillus Bifidobacterium V9 (Kex02) Improving the Efficacy of Carilizumab Combined With Platinum in Non-small Cell Lung Cancer Patients	Recruiting	Not available	Non-Small Cell Lung Cancer	*Bifidobacterium* and *Lactobacillus*; Placebo	China	https://clinicaltrials.gov/ct2/show/NCT05094167
Effect of Live Combined *Bifidobacterium*, *Lactobacillus* and *Enterococcus* Capsules on Oral Mucositis in NasopharyngealCarcinoma Patients Receiving Radiotherapy	Unknown status	Not available	Oral mucositis in Nasopharyngeal Carcinoma	*Lactobacillus*, *Bifidobacterium* and *Enterococcus*	China	https://clinicaltrials.gov/ct2/show/NCT03112837

## BIFIDOBACTERIUM AND THE IMMUNOCHEMOTHERAPY LANDSCAPE

In this context, *Bifidobacterium* deserves special attention as it has been directly involved in the response to immunochemotherapy^[63,64]^. For example, in 2015, Sivan *et al.* compared antitumor T lymphocyte responses in murine models purchased from two different facilities^[65]^, namely Jackson Laboratory (JAX) and Taconic Farms (TAC) mice. Compared to TAC mice, JAX mice developed adequate antitumor immunity. Interestingly, the mice also differed in GM composition, with *Bifidobacterium* being overabundant in JAX mice. After induction of melanoma in both murine models, JAX mice exhibited reduced tumor cell growth and enhanced T cell-mediated immune surveillance. Overabundance of *Bifidobacterium*, particularly species *B. breve*, *B. longum* and *B. adolescentis*, was positively associated with the antitumor response mediated by activation of T cell effectors. Furthermore, oral administration of *B. breve* and *B. longum* to mice with *Bifidobacterium* depletion was sufficient to reduce natural melanoma growth alone and, in association with immunotherapy (anti-PD-L1), to restore specific antitumor T cell responses and almost abolish tumor outgrowth. Increased dendritic cell function leading to T cell activation in the tumor microenvironment was shown in mice receiving *B. breve* or *B. longum*, compared to germ-free mice or mice natively without *Bifidobacterium* in the GM. These findings were validated in a cohort of patients with metastatic melanoma, where metagenomic sequencing revealed pre-treatment enrichment of *B. longum*, along with *A. muciniphila*, *Collinsella aerofaciens*, and *Enterococcus faecium*, in those who responded to anti-PD-L1 immunotherapy^[46]^. Furthermore, fecal microbiota transplantation into germ-free mice with responder GM led to the restoration of anti-PD-L1 treatment efficacy. A study by Rong *et al.* suggested that *Bifidobacterium* may potentiate the antitumor immune response by promoting the long-term survival of CD8+ T cells as memory cells and thus stimulating the immune response in some immunotherapy treatments^[66]^. In another recent study, it was demonstrated that *Bifidobacterium* strains induce potent CD8+ T cell-mediated antitumor immunity in animal models, thus enhancing the treatment with immune checkpoint inhibitors^[67]^. Interestingly, several years earlier, Li *et al.* suggested a direct role of *Bifidobacterium* spp. at the tumor site^[68]^, by demonstrating their translocation from the gastrointestinal tract into the bloodstream and selective accumulation in tumors, due to their ability to survive in the hypoxic, nutrient-rich environment created by tumor cells^[69]^. Furthermore, the potential of *B. adolescentis* as a highly specific vector for the transport of anticancer genes to a target tumor has been demonstrated. In murine models subcutaneously implanted with liver cancer cells, intravenous injection of *B. adolescentis* previously transformed to express a gene encoding the antiangiogenic protein endostatin resulted in germination and proliferation of microbes within the tumor bed, but not in non-malignant tissues. In addition, intratumoral expression of endostatin and thus inhibition of tumor growth were observed.

On the other hand, evidence is also accumulating on a not entirely favorable role of *Bifidobacterium* in the response to immunochemotherapy. For example, *Bifidobacterium* were found to be overabundant in the GM of platinum-resistant patients treated for epithelial ovarian cancer^[70]^. As speculated by the authors, the lactate produced by these microbes as part of their metabolism could fuel the “Warburg effect” (i.e., the production of lactate by aerobic glycolysis)^[71,72]^. Increased lactate production is frequently observed in tumor cells, where it promotes angiogenesis, tumor growth, inflammation, metastasis, epithelial-mesenchymal transition and immune evasion. The hypothesis put forward by the authors is therefore that *Bifidobacterium*, and potentially other lactic acid bacteria, are capable of interfering with the lactate cycle, increasing its local and systemic bioavailability and thus influencing tumor progression, as well as the efficacy of chemotherapy. While fascinating, it should be noted that these speculations are based on the detection of increased proportions of *Bifidobacterium* and other potential lactate producers (and predicted lactate production pathways) while decreased proportions of lactate utilizers in platinum-resistant *vs.* platinum-sensitive patients, without direct measurement of lactate levels (and its isoforms). In this regard, the lactate levels typically produced in the intestine are much lower than those obtained at the tumor site by the Warburg effect^[72]^, further underlining the need to verify the proposed link. Furthermore, it cannot be ruled out that GM alterations in potential lactate producers/utilizers are only a side effect of chemotherapy, in combination with other host factors, with no direct role in treatment response. However, there is previous evidence that similarly demonstrated an increase in lactic acid bacteria, including *Bifidobacterium*, in patients with gastrointestinal cancer, and suggested a role for them in influencing tumor development, also through exogenous lactate supply^[73]^. Once again, these are purely associative observations, which makes proof of concept mandatory, especially in extra-intestinal cancers.

In support of the latter speculations, it should be mentioned that *Bifidobacterium* has a known anti-inflammatory role, mediated by the production of SCFAs and induction of Treg cells and IL-10, so it is not entirely unreasonable to doubt its ability to promote antitumor immune responses. Perhaps the discriminating factor, as the studies commented above suggest, is the use of immune *vs.* chemotherapy protocols. In the context of immunotherapy, we hypothesize that *Bifidobacterium*-induced Treg cells in the intestine might migrate into the tumor microenvironment and promote tumor cell evasion mechanisms from immunosurveillance. In this regard, in a very elegant and recent study in murine models, Fidelle *et al.* revealed that enterotropic T cells can actually relocate to distant tumors and impair the therapeutic efficacy of immune checkpoint inhibitors^[74]^. Notably, such a relocation was favored by gut dysbiosis-induced downregulation of the expression of mucosal addressin cell adhesion molecule 1 (MAdCAM-1) on intestinal endothelial cells, which usually helps retain an immunosuppressive set of T cells (Treg17 cells) within the gut, through interaction with integrin 47. Disruption of MAdCAM-1-47 interaction triggered the migration of Treg17 cells from the ileal lamina propria and gut-associated lymphoid tissues to distant tumors and tumor-draining lymph nodes, where they impaired the therapeutic efficacy of immune checkpoint inhibitors by producing immunosuppressive molecules such as IL-10, CD39 and CD73. Moreover, the tumor microenvironment was found to be associated with metabolic reprogramming of tumor-infiltrating Treg cells, which increases their reliance on fatty acid (instead of glucose) metabolism, further contributing to the Warburg effect^[75]^. However, the process of Treg cell migration to tumor sites and the interaction of migrated immune cells with cancer cells (including factors driving their metabolic behavior) are highly complex processes not yet fully understood. More evidence is needed to better elucidate the role of gut-primed Tregs in cancer immunosurveillance and whether/how *Bifidobacterium* (or other GM components) is involved in this intricate picture.

## GUT MICROBIOME-BASED ANTICANCER INTERVENTION STRATEGIES INVOLVING *Bifidobacterium* SPP

The results of clinical and preclinical studies investigating the functional roles of *Bifidobacterium* spp. in the cancer setting have so far been sadly inconclusive. However, the use of *Bifidobacterium* as a probiotic, alone or in combination with *Lactobacillus* spp., has led to numerous benefits in multiple pathological contexts^[76]^. With specific regard to cancer, the intake of *Bifidobacterium* spp. conferred protection against CRC development in mice^[77]^ and improved immune function in CRC patients^[78]^. Furthermore, *Bifidobacterium* mitigated the secondary effects of surgery and chemotherapy in patients with CRC, as well as in those undergoing colectomy or resection of liver metastases^[79,80]^. In particular, *B. breve* reduced post-chemotherapy GM dysbiosis and limited the development of infections in a pediatric cohort^[81]^. In contrast, another study focused on head and neck cancer patients treated with a cocktail of *Bifidobacterium* spp. and *Lactobacillus* spp. showed no improvement in patients’ clinical outcomes (i.e., inflammatory markers and gut permeability)^[82]^. However, it should be mentioned that these conflicting results may be partly explained by interindividual variations in GM and host genomes. Indeed, several studies confirmed that the intestinal colonization and functionality of probiotics are strongly influenced by the individual GM, the host gene expression profile, and other exogenous factors^[83-85]^.

Notwithstanding the above, a number of clinical trials have been planned (some of which are still ongoing) to examine the therapeutic potential of *Bifidobacterium* interventions in cancer patients. A list of clinical studies registered in the previous two years is shown in Table 1. In an ongoing interventional randomized study of 30 participants with advanced liver cancer receiving immunotherapy, Xie *et al.* are profiling the GM of patients receiving or not a cocktail of lactic acid bacteria, including *Bifidobacterium* spp. (NCT05620004). Another interventional randomized clinical trial was designed to evaluate the impact of oral administration of *Lactobacillus* and *Bifidobacterium* on 46 participants with NSCLC on the efficacy of immunochemotherapy (NCT05094167). Other ongoing studies are investigating the role of *Bifidobacterium* in improving the conditions of patients before and after cancer removal surgery, by mitigating therapy-related side effects^[86,87]^. Furthermore, in a randomized controlled clinical trial of 180 patients with hepatocellular carcinoma (NCT05178524), investigators used *Bifidobacterium*-rich BIFICO as an intervention strategy to sustain medication in the perioperative period of hepatectomy and observed postoperative liver functionality recovery. The first published research on this project revealed that the GM profile influenced the rate at which liver function recovered after hepatectomy, and, again, *Bifidobacterium* was a major player. In an interventional randomized clinical trial (NCT03112837), investigators evaluated the impact of combined live capsules of *Bifidobacterium*, *Lactobacillus* and *Enterococcus* in patients with nasopharyngeal carcinoma. In particular, the aim of this study was to determine whether GM modulation reduced the severity of radiation-induced mucositis in patients receiving radical dose radiotherapy. Indeed, radiation-induced mucositis is an acute mucosal reaction of patients undergoing head and neck radiotherapy, which leads to dose-limiting and debilitating side effects.

## CONCLUSIONS AND FUTURE TRENDS

GM has now taken a leading role in research focused on maintaining host well-being, including the success of anticancer therapies. In this scenario, several works have linked *Bifidobacterium* to improved response to immunochemotherapy. However, its well-known anti-inflammatory and immunosuppressive properties and its recent association with platinum resistance do not allow definitive conclusions to be drawn. Nevertheless, some clinical trials are already underway involving the intake of *Bifidobacterium* directly in cancer patients. It goes without saying that further studies in large cohorts are needed to fill the many knowledge gaps and provide robust experimental evidence. Such studies should possibly be conducted with different omics approaches (including metabolomics) and animal models to finally go beyond simple associations and uncover the underlying molecular mechanisms. Only the achievement of such a goal will truly allow precision strategies to be implemented. In the near future, it is possible to foresee not only that *Bifidobacterium*l strains will be rationally used to induce certain anticancer effects in a given context, but that they will eventually be engineered to either boost or hinder some functionalities instrumental to the success of immunochemotherapy protocols. Not least, it is possible to envisage the use of *Bifidobacterium*-derived postbiotics^[88]^ to specifically confer beneficial effects by directly using the molecular actors involved. This could be particularly relevant in the case of cancer patients, often immunocompromised and with an increased risk of infections and sepsis.

## DECLARATIONS

### Copyright

© The Author(s) 2023.
